# Supplementary material for: Human agency and infection rates: Implications for social distancing during epidemics
Source: PLoS One. 2020 Dec 15;15(12):e0243699. doi: 10.1371/journal.pone.0243699 (PMC7737959; doi:10.1371/journal.pone.0243699)
Supplement: S1 File — (HTML) [file pone.0243699.s001.html]

Epidemic Model


# Interactivity-based epidemic model

**doi:** https://doi.org/10.1371/journal.pone.0243699

---

|  |  |  |  |
| --- | --- | --- | --- |
| r0 |  | 95% t variation factor |  |
| Maximum t/t\_median |  |  |
| t\_bar |  | t2\_bar |  |
| t\_median |  | Activity ratio |  |
| Steps |  | Response delay |  |
| Seed proportion |  | Final case rate |  |
| Group proportions |  | Group colors |  |
| Group mortality |  | Overall mortality |  |
| Group measures |  | Group thresholds |  |
| Group factors |  | Use factors |  |
| Run | No intervention Protection Mitigation Suppression Lockdown 1 Lockdown 2 Elimination | | |
| Export plot | Cycle data Quantile data Summary data JSON data | | |

---


---

A) case rate per cycle as a proportion of population (overall shown in black); B) cumulative case proportion in each group and for the population as a whole; C) underlying Reff value for measures employed (purple) and relative interactivity for population (brown); D) final distribution of infections as a function of interactivity quantiles of the population by group.

---

## Help

### The theoretical model parameters

This has three parameters to set it up. These are:

- R0: the natural growth rate of the epidemic per cycle.
- 95% t variation factor: this is the variation in interactivity in individuals.
- Maximum t/t\_median: this is a maximum interaction level for individuals, expressed as a factor from the median.

Setting these three will calculate t\_bar, t2\_bar (the mean values of t and t squared) and t\_median.

### Scenario testing

The following are the set parameters for scenarios:

- Steps: this sets the number of steps to calculate over.
- Response delay: this is the number of cycles after an infection rate has been reached, when measures are put in place.
- Seed proportion: this is the proportion of the population infected in cycle 0.
- Group proportions: this array sets up the number of groups within the model; the numbers should add to 1.
- Group colors: these are used for plotting.
- Group mortality: this should be an array giving the mortality rate of cases for each group.
- Group measures: this is an array of arrays (top level groups), with the caps to activity, in standard deviations from the median t value (using a log-normal distribution), for each of the thresholds.
- Group thresholds: these are the case-rate thresholds at which the measures are applied.
- Group factors: this is an alternative to using caps on activity, and applies a reduced interactivity factor on the groups.
- Use factors: this is the option to use factors rather than caps.

The model will output three summary parameters:

- Activity ratio: this is the factor by which the overall interaction between individuals in the population is reduced.
- Final case rate: this is the total proportion of infected individuals in the population.
- Overall mortality: this is the mortality proportion of the population from the infections gained within the model period.

### Pre-set scenarios

The scenarios described in the paper are preset and can be selected by the buttons.

### Data output

Figures are generated for the model runs within the web application. These can be exported as SVG files using the [Export plot] button.

The data from the model runs can be extracted as plain text using the [Cycle data], [Quantile data] and [Summary data] buttons. Alternatively a structured JSON object with all the data can be exported using the [JSON data] button.

---
